# Severity of Idiopathic Scoliosis Is Associated with Differential Methylation: An Epigenome-Wide Association Study of Monozygotic Twins with Idiopathic Scoliosis

**DOI:** 10.3390/genes12081191

**Published:** 2021-07-30

**Authors:** Patrick M. Carry, Elizabeth A. Terhune, George D. Trahan, Lauren A. Vanderlinden, Cambria I. Wethey, Parvaneh Ebrahimi, Fiona McGuigan, Kristina Åkesson, Nancy Hadley-Miller

**Affiliations:** 1 Musculoskeletal Research Center, Children’s Hospital Colorado, Aurora, CO 80045, USA; patrick.carry@cuanschutz.edu; 2 Department of Orthopedics, University of Colorado Anschutz Medical Campus, Aurora, CO 80045, USA; elizabeth.a.terhune@cuanschutz.edu (E.A.T.); cambria.wethey@cuanschutz.edu (C.I.W.); 3 Department of Pediatrics, University of Colorado Anschutz Medical Campus, Aurora, CO 80045, USA; devon.trahan@cuanschutz.edu; 4 Department of Biostatistics and Informatics, Colorado School of Public Health, Aurora, CO 80045, USA; lauren.vanderlinden@cuanschutz.edu; 5 Clinical Sciences Malmo, Clinical and Molecular Osteoporosis Research Unit, Lund University, S-205 02 Malmö, Sweden; parvaneh.ebrahimi@med.lu.se (P.E.); fiona.mcguigan@med.lu.se (F.M.); kristina.akesson@med.lu.se (K.Å.); 6 Department of Orthopedics, Skane University Hospital, S-205 02 Malmö, Sweden

**Keywords:** idiopathic scoliosis, monozygotic twin, epigenome-wide association study, DNA methylation, bone, discordant, curve severity, differentially methylated region

## Abstract

Epigenetic mechanisms may contribute to idiopathic scoliosis (IS). We identified 8 monozygotic twin pairs with IS, 6 discordant (Cobb angle difference > 10°) and 2 concordant (Cobb angle difference ≤ 2°). Genome-wide methylation in blood was measured with the Infinium HumanMethylation EPIC Beadchip. We tested for differences in methylation and methylation variability between discordant twins and tested the association between methylation and curve severity in all twins. Differentially methylated region (DMR) analyses identified gene promoter regions. Methylation at cg12959265 (chr. 7 *DPY19L1*) was less variable in cases (false discovery rate (FDR) = 0.0791). We identified four probes (false discovery rate, FDR < 0.10); cg02477677 (chr. 17, *RARA* gene), cg12922161 (chr. 2 *LOC150622* gene), cg08826461 (chr. 2), and cg16382077 (chr. 7) associated with curve severity. We identified 57 DMRs where hyper- or hypo-methylation was consistent across the region and 28 DMRs with a consistent association with curve severity. Among DMRs, 21 were correlated with bone methylation. Prioritization of regions based on methylation concordance in bone identified promoter regions for *WNT10A* (WNT signaling), *NPY* (regulator of bone and energy homeostasis), and others predicted to be relevant for bone formation/remodeling. These regions may aid in understanding the complex interplay between genetics, environment, and IS.

## 1. Introduction

Adolescent idiopathic scoliosis (IS) is a three-dimensional spinal deformity affecting 1–3% of otherwise normal prepubescent and adolescent individuals [1,2]. Screening programs, conservative treatment, and surgical care in the case of progressive curvatures impose significant personal, familial, financial, and societal costs across the lifetime of affected individuals. The etiology of IS remains unknown. However, it has been shown to have a strong familial component [3] with a sibling recurrence risk of 18%, and heritability estimates of approximately 87.5% [4,5,6].

Traditional genetic association methods including familial linkage studies [7,8,9,10,11,12,13,14,15,16,17], exome sequencing [18,19,20,21,22,23,24,25,26,27], and genome wide association studies (GWAS) [28,29,30,31,32,33,34,35,36] have resulted in a number of positive associations with IS, of which only a few loci, notably those in or near *ADGRG6* [31,37,38,39,40,41,42,43] and *LBX1* [21,29,30,44,45,46,47,48,49,50,51,52,53,54,55,56,57], have been replicated across multiple independent study populations [58,59]. While familial and case–control designs have added to our understanding of IS, the complex heterogenic nature of IS [58,59,60,61,62] has limited our understanding of the genetic underpinnings of this particular disorder. The combination of our inability to relate specific genetic variants to the biology of IS, the low reproducibility of results, increased prevalence of more severe curves among females [63], and the wide variation in phenotype has increased interest in the potential role of environmental and/or epigenetic factors in the etiology of IS [59,64,65].

One frequently studied mechanism of epigenetic regulation is DNA methylation in which a methyl group is added to the cytosine nucleotide within a DNA sequence. Typically occurring in a cytosine phosphate guanine (CpG) dinucleotide pair, methylation has the capacity to change chromatin structure and alter transcription factor binding [66]. This is a reversible event that may provide a link between genetic variation, environment, and disease [67,68]. Although tissue-specific, up to 80% of the variation in the epigenome may be due to genotype [69], therefore a challenge among epigenome wide association studies (EWAS) is determining whether the observed epigenetic phenotype association is due to environmental or genetic effects. Studying monozygotic (MZ) twins, is one way to minimize this concern. MZ twins discordant for the phenotype of interest are near perfect genetic matches, therefore their DNA methylation levels can be compared to shed light on the phenotypic expression of the disease.

Previous epigenome-wide association studies have provided evidence supporting the role of DNA methylation in numerous complex musculoskeletal diseases including osteoarthritis [70], osteoporosis [70], cerebral palsy [71], and Paget’s disease of bone [72]. The role of DNA methylation in IS has not been well studied. Targeted studies have reported associations between methylation and IS near the *COMP* [73] and *PITX1* [74] genes. Two recent studies [75,76] of *ESR1* and *ESR2* methylation from paravertebral muscle tissue in females with IS supports potential interrelationship between sex hormone levels, methylation, and the clinical manifestation of IS. *ESR1* methylation levels from paravertebral muscle tissue on the concave side of curve were associated with curve severity [75], and furthermore, *ESR2* promoter methylation levels differed between concave and convex sides of the curves [76]. Epigenome-wide discovery analyses in MZ twins are limited to studies including only one [77] and two [78] MZ twin pairs discordant for IS. There is a strong need for additional epigenome wide analyses to understand the potential role of DNA methylation in IS. Therefore, the aim of this EWAS was to identify differences in DNA methylation levels between monozygotic twin pairs discordant for IS. Within twin pairs, we also aimed to determine if differences in methylation were associated with differences in curve severity.

## 2. Materials and Methods

### 2.1. Study Population

Peripheral whole blood samples were obtained from 8 female monozygotic twin pairs (*n* = 16 individuals) diagnosed with idiopathic scoliosis (IS). Participants were identified from an existing registry, the Genetics of Idiopathic Scoliosis project (the GenesIS project), that has been described by Baschal et al. [19]. A diagnosis of IS required that subjects had no congenital deformities or other co-existing genetic disorders and a standing anteroposterior radiograph showing a curvature of at least 10° by the Cobb method [79]. There were 6 twin discordant twin pairs (difference in primary curve Cobb angle >10°) and 2 concordant twin pairs in our study population. The difference in the primary curvature among the two concordant twin pairs was <1 and 2°, respectively (Table 1). Written informed consent and assent, when appropriate, was obtained from all study participants and/or their legal guardians in accordance with protocols approved through the Johns Hopkins School of Medicine Institutional Review Board and the University of Colorado Anschutz Medical Campus Institutional Review Board.

### 2.2. DNA Methylation Processing

Genomic DNA was extracted from fresh whole blood using a standard phenol-chloroform purification procedure [80]. DNA was further purified using the Zymo DNA Clean & Concentrator kit, followed by Nanodrop quantification. Approximately 1 µg DNA was bisulfite converted using the Zymo EZ DNA Methylation kit (Zymo Research, Irvine, CA, USA). The precipitated DNA was dispensed onto the Infinium MethylationEPIC 850 K BeadChip (Illumina). The EPIC chip provides methylation measurements across the genome. All sequencing was performed at the University of Colorado Genomics and Microarray Shared Resource. Twin pairs were consistently arranged in sequential order on the plate to minimize within and between batch effects.

The 850 K Infinium platform includes 866,836 annotated probes representing individual cytosine phosphate guanine (CpG) probe sites. Data were normalized using the SWAN normalization method implemented within the minfi R package [81]. Standard quality control checks were performed at both the sample and probe level using the minfi R package [81]. Probes or samples that failed any of the following standard filtering procedures were treated as missing data: probe was not detectable above background noise (*n* = 776 [<0.1%]), probes with a low bead count (>5% of samples with a beadcount <3) (*n* = 17,272 [2%]), cross reactive probes (*n* = 17,028 [2%]), dropped during initial quality control processing (*n* = 528 [<0.1%]). Probes located on sex chromosomes (*n* = 15,648 [1.8%]) and probes that included known SNPs (*n* = 165,678 [19.1%]) were also excluded from the analysis. Consistent with recommendations of Logue et al. [82], we filtered low variability probes known to be associated with poor reproducibility. We filtered out all probes with β range value < 0.05%, *n* = 145,163 (16.7%). In total, 504,743 probes met the inclusion criteria and were included in subsequent steps.

The microarray methylation measurements were performed in two batches. The ComBat function implemented in the sva R package [83] was used to adjust the SWAN normalized M values for potential batch effects. The batch adjusted M-values were used in all statistical analyses. The current Illumina annotation for the 850 K platform is on hg19. Only probes which match 100% to a single location were used for further analyses.

**Figure 1:**
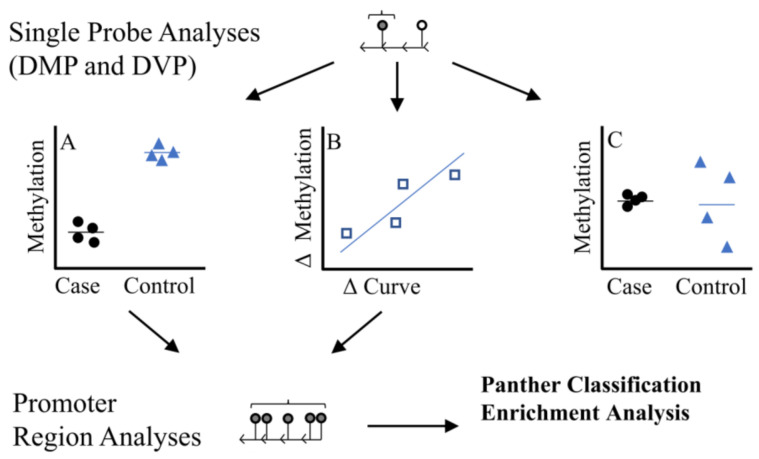
Study Methodologic Workflow. Differentially methylated position (DMP) analyses were used to identify single probes. We used two DMP strategies (1) Discordant DMP Analysis (**A**), differences in methylation between cases (twin with more severe IS) relative to controls (twin with less sever IS) (2) Severity DMP Analysis (**B**) looked at association between difference in curve severity and methylation ${∆}_{curve}vs\;{∆}_{methylation}$, where ${∆}_{methylation}=$ methylation levels in affected/more severe twin–methylation levels unaffected/less severe twin and ${∆}_{curve}=$ primary curve magnitude in affected/more severe twin–primary curve magnitude is unaffected/less severe twin. We also looked at differences in variability at single probes (differentially variable position analysis, DVP) among cases compared to controls (**C**). Region analyses based on single probes from (**A**,**B**) were used to identify regions of consistent methylation effects within promoter regions. We only considered regions with 5 or more probes where direction of effect was consistent across all probes. DVP probes (**C**) were not considered in the region analysis due to challenges interpreting a ‘consistent’ direction of effect based on variability.

We used three analytical approaches to identify individual CpG sites (Figure 1): (A) we performed a discordant differentially methylated position (DMP) analysis, testing for differences in methylation (batch adjusted M-values) between six twin pairs where the difference in the primary spinal curvature Cobb angle was >10°, (B) Using all twins, we tested the association between within twin pair differences in methylation (${∆}_{methylation}=$ methylation levels in affected/more severe twin–methylation levels unaffected/less severe twin) and differences in curve severity within twin pairs (${∆}_{curve}=$ primary curve magnitude in affected/more severe twin–primary curve magnitude is unaffected/less severe twin), (C) We tested for differences in methylation variability between the discordant twin pairs (Differentially Variable Position [DVP] analysis). The methods workflow is outlined in Figure 1.

Methylation is tissue specific. Confounding due to differences in cell composition between cases and controls is a potential concern in epigenome-wide association studies [84]. Cell proportions were estimated from methylation values using the minifi [81] R package. The distribution of CD8T, CD4T, B Cells, natural killer Cells, monocytes, and neutrophils was similar in cases compared to controls (Appendix A, Table A1). Due to the small sample size and similar distribution of cell proportions across all individuals, we did not adjust for cell type in subsequent analyses.

### 2.3. Statistical Analysis

We used descriptive statistics to summarize the demographic and clinical characteristics of all subjects included in the study. Wilcoxon signed rank tests were used to compare the distribution of the five cell types between case and control twins (Table 1 and Appendix A, Table A1). Normalized M-values were used in all analyses. β-values and percent methylation were also reported to facilitate biological interpretation. In the discordant DMP analysis, paired t-tests were used to test for differences in M values between discordant twins (*n* = 6 pairs, *n* = 12 individuals). The individual with the more severe curvature was designated as the “case”, and the corresponding twin with the less severe curvature was designated as the “control.” For the DVP discordant analysis, a regularized version of Bartlett’s test was used to identify differentially variable probes between discordant twin pairs. For the curve severity DMP analysis involving all twin pairs, linear regression models were used to test the association between differences in methylation (${∆}_{methylation}$) and differences in curve severity within twin pairs (${∆}_{curve}$). To account for multiple testing, false-discovery rate (FDR) adjusted p values were estimated using the algorithm described by Benjamini and Hochberg [85].

An exploratory analysis was used to identify differentially methylated regions (DMR) using the mCSEA [86] R package among promoter regions with a minimum of 5 probes. Significance was assessed based on 100,000 permutations. The DMR analysis was implemented for the discordant DMP and the curve severity DMP analyses. Based on the exploratory nature of the DMR analysis, only regions where 100% of probes were in the same direction of effect and the FDR adjusted *p* value was < 0.05 were considered significant.

Functional gene overrepresentation analysis was performed based on the results of the discordant DMP and curves severity DMP analyses. The top DMRs, nominal *p* value <0.001, were included in the DMR overrepresentation analysis. Overrepresentation analyses of the severity and discordant gene lists were conducted using PANTHER v16.0, test release 20,210,224 (PantherDB.org) [87,88,89]. Custom gene background inputs were used in accordance with gene promoter region DMRs analyzed within the Infinium HumanMethylation EPIC Beadchip platform. The Annotation Data Sets Gene Ontology (GO) Cellular Component Complete, GO Molecular Function Complete, and GO Biological Process Complete were analyzed separately, each using a Fisher’s Exact Test and Bonferroni correction. We report overrepresented GO terms with a Bonferroni-adjusted *p* < 0.05. Significant terms were reviewed; parent terms were manually removed and REVIGO [90] was used to eliminate redundant terms.

### 2.4. Prioritization of Candidates: Blood Methylation Correlation

While the tissue of origin for IS has not been determined, the primary clinical manifestation is that of the bony spinal column. Ebrahimi et al. [91] previously reported on methylation levels in whole blood versus trabecular bone. We utilized these data to prioritize our methylation candidates as ones that may have a functional role in bone. We reviewed the distribution of correlation coefficients, representing the strength of association between methylation levels in blood versus bone, among all probes evaluated by Ebrahimi et al. [91] Probes were considered strongly correlated if the correlation coefficients exceeded the 75th percentile among all probes evaluated by Ebrahimi et al. [91] (ρ = 0.49). We then reviewed the correlation coefficients for probes identified as candidates in our DMP and DMR analyses. For the DMR analysis, we reported the maximum correlation coefficient among all probes in the DMR as well as the percentage of strongly, positively correlated probes across the entire region.

## 3. Results

### 3.1. Demographics

The study population included 6 discordant and 2 concordant female monozygotic twin pairs with idiopathic scoliosis (IS, see Table 1). The average age among all individuals at the time of blood acquisition was 37.3 years (±22.5). The average Cobb angle of the primary curve was 39.6° (±15.3). The average difference in age between twin pairs at the time of sample acquisition was 1.2 months (range: 0 to 6 months). The average difference in curve severity among all twins was 19° (range: 0 to 44°). Among discordant twins, the average difference in curve severity was 25° (range: 11 to 44°).

### 3.2. Discordant Curvature Analysis

**Figure 2:**
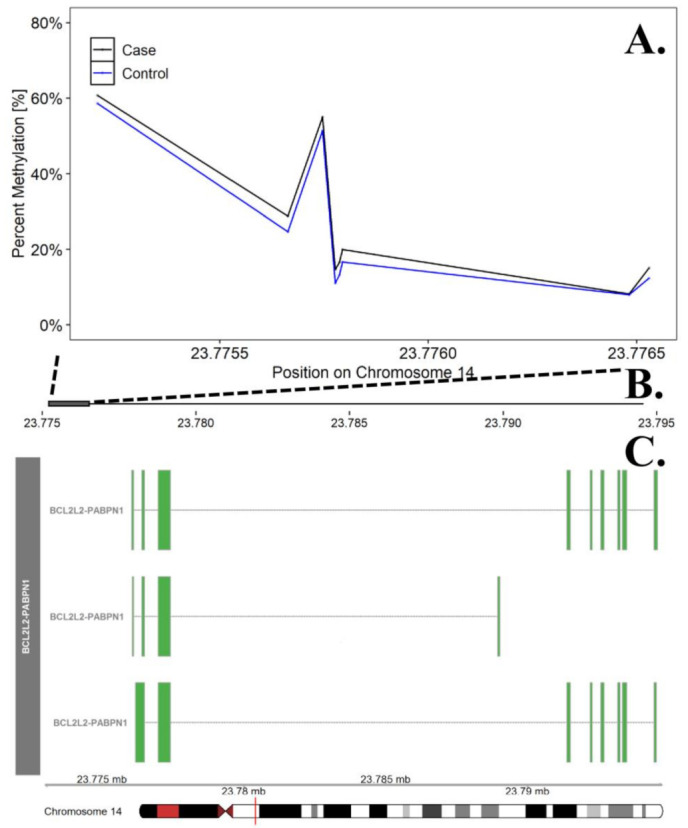
Differentially Methylated Region in the *BCL2L2-PABN1* Promoter Region on Chromosome 14. The top panel (**A**) describes differences in percent methylation between cases and controls at each probe included in the promoter region for *BCL2L2-PABN1*. This region was the most significant DMR in the discordant twin analysis. The X axis represents the position (mb) of the probes. The middle panel (**B**) represents the location of promoter region (solid square) relative to the entire gene, represented in the bottom panel. Multiple known isoforms of *BCL2L2-PABN1* are represented in the bottom panel (**C**), boxes represent exons and lines represent introns. The red line on the ideogram, bottom of the figure, represents location of the region within the chromosome.

In the discordant analysis, none of the individual CpG probes were significant at the FDR adjusted *p* = 0.10. Differentially methylated region (DMR) analyses identified 200 promoter regions (containing 5–14 CpG sites) that were significant at the FDR adjusted *p* value of 0.05. Among these regions, 58 DMRs included probes/sites where the direction of effect (hypermethylation or hypomethylation) was consistent across 100% of the probes (Appendix A, Table A2). The most significant DMR represented a region on chr. 14 in the promoter region for the *BCL2L2-PABPN1* gene (FDR adjusted *p* = 0.0113, see Figure 2). Using the Panther enrichment algorithm with these 58 DMRs, we identified 1 significantly enriched gene ontology (GO) term (Appendix A, Table A3).

**Figure 3:**
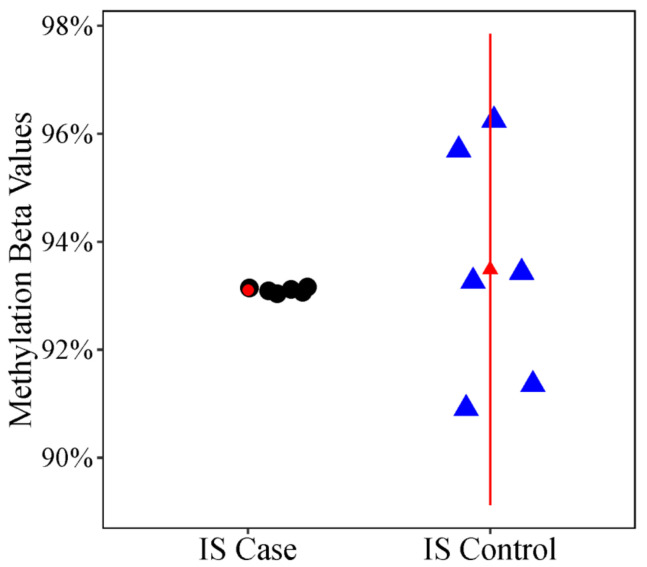
Scatter Plot of Differential Variability Between Case and Control Twins. Methylation levels (% methylation or β values) at the cg12959265 probe, an open sea probe near the *DPY19L1* gene on chromosome 7 in IS cases and IS controls. The triangle represents the mean % methylation and error bars represent +/− 1 standard deviation. The plot illustrates the large difference in variability at cg12959265 in IS cases vs. IS controls.

In addition to differences in methylation levels, we looked for differences in methylation variability, which may provide valuable information about the heterogenous environmental exposures that contribute to disease etiology. In the differentially variable position (DVP) analysis, methylation variability at cg02477677 was significantly lower (FDR adjusted *p* value = 0.0791) in cases with a more severe curve compared to unaffected or less severely affected controls (Figure 3). The cg02477677 CpG probe is an open sea probe on chr. 7 near *DPY19L1*.

### 3.3. Curve Severity Analysis

**Figure 4:**
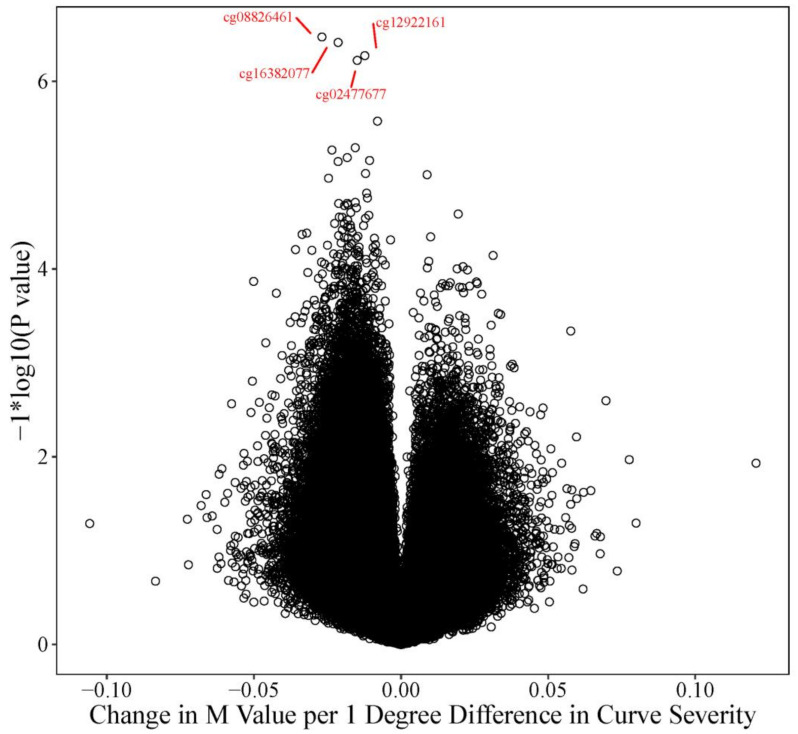
Volcano Plot: Curve Severity Analysis The volcano plot describes the effect size and *p* value for every probe tested in the curve severity analysis. The Y axis represents the −log10(*p* values) and the X axis represents the change in M value for every one-degree difference in curve severity between the twin pairs for each of the respective probes tested. Increasing curve disparity was more often associated with hypomethylation (decreased M values, left or negative side of the plot) than hypermethylation. The four FDR significant probes (FDR adj *p* = 0.0753) are highlighted in red, cg08826461 (nominal *p* value = 3.37 × 10^−7^), cg16382077 (nominal *p* value = 3.85 × 10^−7^), cg12922161 (nominal *p* value = 5.32 × 10^−7^), and cg02477677 (nominal *p* value = 5.97 × 10^−7^).

We also tested whether the difference in methylation between cases and controls was associated with the difference in curve severity between cases and controls. We identified 4 CpG sites where the difference in methylation was significantly associated (FDR adjusted *p* value = 0.0753) with the difference in curve severity (Figure 4). At each of these open sea probes, increasing disparity in curve severity between cases and controls was associated with a pattern of hypomethylation.

For every 1 degree increase in the difference in curve severity in cases compared to controls, batch adjusted M-values decreased by an average of between 0.012 to 0.027 units. Significant probes included cg02477677 (slope: 0.015 units, near the *RARA* gene on chr. 17, nominal *p* value = 5.97 × 10^−7^); cg08826461 (slope: 0.027 units chr. 2, does not map to a known gene, nominal *p* value = 3.37 × 10^−7^), cg12922161 (slope: −0.012 chr. 2, maps to *LOC150622*, nominal *p* value = 5.32 × 10^−7^), and cg16382077 (slope: 0.021 units, chr. 7, does not map to a known gene, nominal *p* value = 3.85 × 10^−7^).

**Figure 5:**
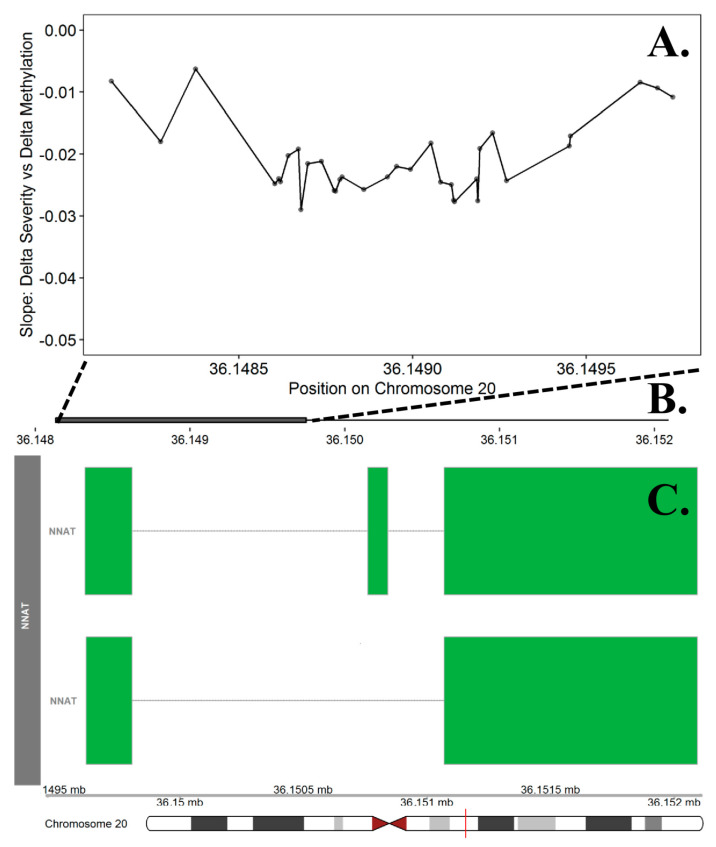
Differentially Methylation Region in the *NNAT* Promoter Region on Chromosome 20. The top panel (**A**) presents the slope estimates from the curve severity analysis that represent the change in methylation between cases and controls per one-degree change in curve severity at each of the 34 probes included in the promoter region for the *NNAT* gene. This region was the most significant DMR in the curve severity analysis. The X axis represents the position (mb) of the probes. The middle panel (**B**) represents the location of promoter region (solid square) relative to the entire gene, represented in the bottom panel. Multiple known isoforms of the *NNAT* gene are represented in the bottom panel (**C**), boxes represent exons and lines represent introns. The red line on the ideogram, bottom of the figure, represents location of the region within the chromosome.

The differentially methylated region analyses identified n = 197 promoter regions (ranging from 5 to 34 CpG sites) significant at the FDR adjusted *p* value of 0.05. Among these, 28 regions included probes where the direction of effect (difference in curve severity was either positively or negatively associated with the difference in methylation between twin pairs) was consistent across 100% of the probes (Appendix A, Table A4). The top DMR consisted of 34 probes on chr. 20 within the promoter region for the *NNAT* gene (FDR adjusted *p* value = 0.0237, Figure 5). Using Panther, we identified 15 significantly enriched ontologies (Appendix A, Table A5). The top biological process terms included pituitary gland development (GO:0021983) and anterior/posterior pattern specification (GO:0009952).

### 3.4. Candidate Prioritization

Ebrahimi et al. [91] conducted an epigenome-wide analysis to measure the correlation between methylation levels in whole blood and trabecular bone. We used these correlation coefficients to prioritize the methylation candidates identified in our study. Among the four probes identified as candidates in our DMP analysis (cg02477677, cg12922161, cg08826461, and cg16382077), only one probe, cg08826461, was strongly correlated with bone tissue (ρ = 0.494, FDR adjusted *p* value = 0.41329). Among DMRs, we prioritized candidate regions where either one or more probes within the DMR was significantly correlated with bone (FDR adjusted *p* value of less than 0.10), or, greater than 50% of probes included in the region were strongly correlated with bone. We identified 13 priority regions based on the discordant DMR analysis and 8 priority candidate regions based on the severity DMR analysis (Table 2).

## 4. Discussions

We utilized an epigenome-wide association study (EWAS) in monozygotic (MZ) twins to identify individual methylation sites and regions across the genome relevant to idiopathic scoliosis (IS). We identified a single CpG site where methylation variability was different between discordant MZ twins and identified CpG sites where increasing curve severity was more often associated with hypomethylation. Differentially methylated region (DMR) analyses identified multiple regions potentially indicative of unique methylation changes within twin pairs discordant for IS as well as unique methylation patterns associated with curve severity. Integration of a peripheral blood/bone methylation dataset allowed us to prioritize regions and sites based on their potential relevance to the IS disease process in bone. Collectively, these results highlight both new and previously reported pathways related to IS curve progression including those involved in neurogenesis and body segmentation.

Differential variability in methylation represents large shifts in methylation that may reflect differential epigenetic and/or environmental effects in cases relative to controls. Differential variability analyses in disease-discordant monozygotic twins have been used to identify DNA methylation signatures associated with Type 1 Diabetes [92] and rheumatoid arthritis [93]. In our analysis, variability at cg0247767, chr. 7 near *DPY19L1,* was significantly different between discordant twin pairs. DPY19L1 is a transmembrane protein localized in the endoplasmic reticulum that regulates neuronal migration and extension during development [94,95]. Zebrafish with mutations within this gene demonstrate spinal axial curvatures [96]; however, the phenotype has not been studied in detail.

We also identified four individual CpG sites that were associated with curve severity (cg02477677, cg12922161, cg08826461, and cg1638077). At these sites, an increase in curve severity tended to be associated with a decrease in methylation (hypomethylation) within the twin pairs. One of the probes, cg12922161, maps to a location near *LOC150622/SILC1*, a non-coding RNA gene. Although the function of this non-coding RNA is not well known, it has been shown to regulate neuron outgrowth and neuroregeneration via cis-acting activation of the transcription factor SOX11 [97]. To date, select non-coding RNAs as non-protein coding regulatory transcripts within the genome have been hypothesized to functionally participate in the initiation and progression of IS [98]. The cg02477677 probe was also associated with curve severity. This probe maps to a region near *RARA* on chr. 17, which encodes a transcription factor for the retinoic acid receptor protein During development, RA signaling plays an essential role in embryonic body axis extension, left-right somite synchronization, and limb development [99]. It is a central mechanism underlying bilateral symmetry during development of the mouse embryo [100]. Right-left asymmetries have previously been hypothesized as a potential contributing factor to IS based on the increased prevalence of IS among individuals demonstrating vestibular and posterior basicranial morphological asymmetries in MRI cross-sectional studies [101,102,103].

Regions of differentially methylated probes (DMRs) may have more important functional implications than methylation levels at a single CpG site, particularly in promoter regions which are areas of the genome where methylation levels tend to be negatively correlated with gene expression [67,68]. Based on the discordant analysis, we identified 58 significant DMRs in known promoter regions, the most significant region included methylation sites with the promoter region for the *BCL2L2-PABPN1* gene on chr. 14. *BCL2L2-PABPN1* is a paralog of *PABPN1*, which is associated with the development of oculopharyngeal muscular dystrophy, a disease characterized by muscular weakness in eyelids, pharyngeal musculature, and limbs [104]. In the curve severity analysis, we identified 28 significant DMRs in known promoter regions, the most significant included probes with the promoter region for the *NNAT* gene on chr. 20. This gene is important for brain development and implicated in neurodegenerative diseases including anterior horn disease [105]. The paternal copy of the *NNAT* gene is exclusively expressed due to imprinting [106,107]. This is potentially relevant to IS given the sex bias of progressive curvatures (females > males), and the higher percentage of affected offspring from paternal IS cases compared to maternal IS cases (80% vs. 56%) [63].

Enrichment analyses of the top DMRs from both the discordant and curve severity results revealed both broad, non-specific ontologies and select ontologies related to neurogenesis, axon guidance and neuron differentiation, all of which can be supported by current literature from both family-based exome sequencing and genome-wide association (GWAS) studies [49,50]. Combined with results in the current study, these data suggests a potential role of neuropathological processes underlying the development and progression of IS.

The complex genetic architecture underlying IS is further complicated by the lack of a clear tissue target. Despite the major clinical manifestation and therapeutic dilemma of the spinal curvature, IS ultimately affects multiple tissue types, one of which is bone. To understand the potential functional relevance of our methylation results in osseous tissue, we reviewed overlap between our identified CpG sites and those reported by Ebrahimi et al. [91] correlating the same methylation markers in matched blood and trabecular bone samples. We identified 21 regions where the DNA methylation in our dataset was correlated with methylation in bone tissue, and therefore, could potentially be considered biologically relevant (Table 2). Annotation of two of the regions implicated the *NPY* gene on chr. 7 and the *WNT10A* gene on chr. 2. The *NPY* gene encodes a neuropeptide expressed throughout the central and peripheral nervous systems [108] and is an essential regulator of bone homeostasis and metabolism [109]. NPY is also a local regulator of osteoblastic lineage and is responsive to mechanical stimuli with potential roles in fracture healing and osteoarthritis [109]. The *WNT10A* gene is a member of the WNT gene class and functions within the WNT10A/β-catenin signaling pathway in regulation of adult epithelial proliferation [110,111], mesenchymal stem cell regulation by stimulating osteoblastogenesis [112], coordination of vertebrate segmentation, and motile cilia function [113].

The role of DNA methylation in IS has not been well studied in current literature outside of two targeted studies and extremely small genome-wide discovery analyses. Mao et al. [73] reported IS cases were associated with increased methylation near the promotor region for the *COMP* gene on chr. 19 and more importantly, decreased COMP expression. Shi et al. [74] identified significantly higher levels of methylation in IS cases versus controls in a region near the pituitary homeobox-1 (*PITX1)* gene, a homeobox transcriptional regulator that plays a role in maintenance of side-to-side musculoskeletal symmetry during development [114]. In our study, methylation levels in the promoter regions for the *PITX1* and/or the *COMP* gene were not differentially methylated in the discordant and/or curve severity analysis. However, pituitary gland development (GO:0021983) and the anterior/posterior pattern specification (GO:0009952) ontologies represented the top 2 most enriched terms in the curve severity DMR analysis (see Appendix A, Table A5).

Meng et al. [78] and Liu et al. [77] conducted the only other IS EWAS studies in the current literature. They used a similar strategy, testing for methylation differences in peripheral blood samples in a discovery cohort (1 and 2 MZ twin pairs, respectively) discordant for curve progression. A second cohort consisting of individuals with IS versus controls was used to confirm methylation sites or regions identified in the discovery cohort. Meng et al. [78] identified a single probe cg01374129 (near the *HSA2* gene) that was significantly hypomethylated in the progressive group compared to the non-progressive group. Liu et al. [77] identified a DMR near the promoter region for the *NDN* gene that was significantly associated with IS. These studies were limited in that the discovery EWAS was performed in a very limited number of individuals. Our study builds on these initial findings with added methylation data from MZ twins both concordant and discordant in their spinal curves. Our complementary analyses provide a list of candidate sites and regions across the genome that may assist in the development of prognostic tools capable of identifying individuals at risk for curve progression. Methylation is tissue specific, we were also able to prioritize hits identified in our analysis based on known correlation with methylation in bone tissue. Additional validation in larger cohorts is needed to confirm these methylation markers as relevant and explore their utility in the clinical setting.

### Limitations

Our study includes several limitations. First, the samples were obtained after disease onset. We cannot exclude the possibility that differences in methylation were caused by changes in curve severity. Although samples within each twin pair were obtained no more than six months apart and thus age at sample acquisition was balanced across the twin pairs, there was substantial heterogeneity in age at sample acquisition across the twin pairs. This is potentially problematic if age modifies the effect of methylation on curve progression. Similarly, we used a case–control design. Cases and controls were defined based on curve pattern information available at the time of sample acquisition. Misclassification of controls is possible if spinal progression occurred over the lifetime of the individual.

## 5. Conclusions

A better understanding of the genetic, epigenetic, and environmental factors underlying IS onset and/or curve progression has significant clinical implications [115,116]. DNA methylation markers may provide value as a prognostic tool for predicting both the initiation and progression of this disorder and furthermore, may also aid in the identification of homogenous subgroups of individuals allowing for more personalized treatment algorithms. In the current study, we identified methylation at specific sites across the genome. Differentially methylation region (DMR) promoter enrichment analyses identified several biologically relevant ontologies related to pituitary gland development, body segmentation and neuronal differentiation. We prioritized the DMR candidates based on known correlation between methylation in blood versus bone. Priority candidates include DMRs in promoter regions related to the WNT signaling pathway (*WNT10A*), a signaling pathway that is relevant to bone formation and remodeling [117], and neuropeptide Y (*NPY*), a regulator of bone and energy homeostasis [109]. This information allows for further targeted studies aimed at understanding the functional relevance of these findings in relation to IS and axial spinal development, alignment, and side-to-side symmetry.

## Figures and Tables

**Table 1:** Demographics and Clinical Characteristics.

Twin Pair	ID	Case Status	Curve Degree ^†^	Age *
Discordant				
1	15505	Case	48	44.8
	15501	Control	41/37	44.8
2	15643	Case	75	81.4
	15642	Control	35	81.9
3	16012	Case	50	16.3
	16009	Control	22	16.3
4	16615	Case	52/48	33.4
	16611	Control	32/28	33.3
5	18453	Case	34	5.6
	18454	Control	23	5.6
6	19294	Case	56/43	48.7
	19292	Control	12	48.7
**Concordant**				
7	16037	NA	45	42.8
	16038	NA	45	42.8
8	18721	NA	26/33	25.3
	18722	NA	29/31	25.5

^†^ Major curve(s), * Age at sample collection.

**Table 2:** Priority DMRs Based on High Correlation with Bone.

Nearest Gene	Chr.	Start Position	End Position	Number of Probes	DMR Nominal *p* Value	DMR FDR *p* Value	Maximum Bone Correlation	FDR Adj. *p* Value for Maximum Bone Correlation	Percent Strongly Positively Correlated Probes Across DMR
**Discordant DMR Analysis**									
*WNT10A*	chr2	219,744,145	219,745,748	9	2.17 × 10^−5^	0.0113	0.83	0.0307	33.3%
*CRISP2*	chr6	49,681,178	49,681,774	11	2.19 × 10^−5^	0.0113	0.89	0.0128	100.0%
*RBPJL*	chr20	43,934,854	43,935,551	12	2.20 × 10^−5^	0.0113	0.72	0.1048	66.7%
*KDM2B*	chr12	122,018,574	122,020,205	14	2.21 × 10^−5^	0.0113	0.83	0.0336	50.0%
*IL27*	chr16	28,518,114	28,519,597	9	4.34 × 10^−5^	0.0156	0.75	0.0844	33.3%
*CA14*	chr1	150,229,143	150,230,345	9	6.51 × 10^−5^	0.0196	0.78	0.0585	33.3%
*C9orf47*	chr9	91,604,473	91,606,140	12	2.64 × 10^−4^	0.0318	0.79	0.0518	18.2%
*STAB1*	chr3	52,528,714	52,529,393	8	3.04 × 10^−4^	0.0329	0.79	0.0534	12.5%
*ACY3*	chr11	67,415,183	67,418,365	8	3.26 × 10^−4^	0.0329	0.72	0.1045	62.5%
*MPG*	chr16	125,896	128,009	11	3.29 × 10^−4^	0.0329	0.82	0.0368	10.0%
*ESM1*	chr5	54,281,198	54,282,459	13	3.97 × 10^−4^	0.0360	0.79	0.0526	61.5%
*TMEM219*	chr16	29,972,752	29,974,294	6	5.57 × 10^−4^	0.0431	0.77	0.0667	66.7%
*CREBBP*	chr16	3,930,112	3,931,489	5	6.16 × 10^−4^	0.0457	0.76	0.0725	80.0%
**Severity DMR Analysis**									
*GANC*	chr15	42,565,522	42,566,390	7	3.38 × 10^−4^	0.0357	0.88	0.0153	28.6%
*NME3*	chr16	1,821,559	1,822,346	8	3.59 × 10^−4^	0.0366	0.76	0.0729	37.5%
*SLC6A5*	chr11	20,619,598	20,621,109	8	3.59 × 10^−4^	0.0366	0.80	0.0489	28.6%
*RAB22A*	chr20	56,883,532	56,885,003	8	4.38 × 10^−4^	0.0391	0.78	0.0594	25.0%
*ACTN4*	chr19	39,137,911	39,138,334	7	4.89 × 10^−4^	0.0407	0.84	0.0298	33.3%
*NPY*	chr7	24,322,873	24,324,570	8	5.58 × 10^−4^	0.0421	0.84	0.0276	37.5%
*RAB38*	chr11	87,908,558	87,909,729	9	6.33 × 10^−4^	0.045	0.73	0.0995	44.4%
*COPB1*	chr11	14,521,639	14,522,617	6	7.79 × 10^−4^	0.0495	0.88	0.0149	50.0%

Maximum Bone Correlation = maximum correlation coefficient representing strength of correlation between blood and bone CpG sites across all sites included in the DMR (from Ebrahimi et al.), FDR *p* Value for Maximum Bone Correlation = FDR adjusted *p* value for maximum correlation coefficient across all sites included in the DMR (from Ebrahimi et al.)., Percent Strongly Correlated Probes Across DMR = Percentage of probes across the entire region where the correlation coefficient representing strength of correlation between blood and bone CpG sites is greater than 75th percentile among all probes tested in Ebrahimi et al.

## Data Availability

Data is available from the author upon reasonable request.

## Appendix A

**Table A1:** Proportion of Blood Cell Populations in IS Cases and Controls.

	Cases			Control			
	Median	Min	Max	Median	Min	Max	*p* Value
CD8T Cell (%)	11.5%	8.3%	33.6%	12.2%	6.9%	18.7%	0.1953
CD4T Cell (%)	18.5%	7.7%	28.3%	14.3%	9.3%	18.0%	0.1953
Natural Killer Cell Count (%)	5.3%	2.2%	8.8%	5.7%	1.9%	8.4%	0.7422
Bcell Count (%)	5.8%	3.5%	10.3%	6.2%	2.9%	10.3%	0.6406
Monocyte Cell Count (%)	7.5%	3.4%	9.5%	8.0%	5.1%	10.9%	0.6406
Neutrophil Cell Count (%)	55.3%	25.7%	67.2%	58.4%	52.0%	72.9%	0.1953

**Table A2:** Analysis of Discordant Curve Severity in Twin Pairs Identified 57 Differentially Methylated Regions where Hyper- or Hypo-methylation was Consistent Across all Probes in the Region.

Feature/Gene	Chr.	Start Position	End Position	Number of Probes	Nominal *p* Value	FDR *p* Value	Maximum Bone Correlation	FDR *p* Value for Maximum Bone Correlation	Percent Strongly Positively Correlated Probes Across DMR
*WNT10A*	chr2	219,744,145	219,745,748	9	2.17 × 10^−5^	0.0113	0.83	0.0307	33.3%
*BCL2L2 **	chr14	23,775,206	23,776,530	8	2.17 × 10^−5^	0.0113	0.50	0.4069	14.3%
*CRISP2*	chr6	49,681,178	49,681,774	11	2.19 × 10^−5^	0.0113	0.89	0.0128	100.0%
*SLFN13*	chr17	33,773,921	33,777,219	11	2.19 × 10^−5^	0.0113	−0.27	0.7380	0.0%
*RBPJL*	chr20	43,934,854	43,935,551	12	2.20 × 10^−5^	0.0113	0.72	0.1048	66.7%
*KDM2B*	chr12	122,018,574	122,020,205	14	2.21 × 10^−5^	0.0113	0.83	0.0336	50.0%
*MS4A3*	chr11	59,822,727	59,828,426	13	2.21 × 10^−5^	0.0113	−0.57	0.2917	7.7%
*GPR21*	chr9	125,794,756	125,797,284	14	2.21 × 10^−5^	0.0113	0.38	0.5940	0.0%
*IL27*	chr16	28,518,114	28,519,597	9	4.34 × 10^−5^	0.0156	0.75	0.0844	33.3%
*AZU1*	chr19	826,359	827,821	9	4.34 × 10^−5^	0.0156	−0.28	0.7263	0.0%
*CA14*	chr1	150,229,143	150,230,345	9	6.51 × 10^−5^	0.0196	0.78	0.0585	33.3%
*HTRA4*	chr8	38,830,814	38,831,857	11	6.58 × 10^−5^	0.0196	0.29	0.7117	0.0%
*ESRP2*	chr16	68,269,763	68,271,177	13	6.62 × 10^−5^	0.0196	0.35	0.6385	0.0%
*ELANE*	chr19	850,975	852,311	8	8.68 × 10^−5^	0.0199	0.45	0.4788	0.0%
*CLDN15*	chr7	100,880,751	100,882,286	10	8.74 × 10^−5^	0.0199	0.68	0.1542	20.0%
*RUNX1*	chr21	36,259,179	36,422,112	14	8.85 × 10^−5^	0.0199	0.60	0.2518	21.4%
*NFE2*	chr12	54,689,278	54,696,210	14	8.85 × 10^−5^	0.0199	−0.36	0.6204	0.0%
*C6orf229*	chr6	24,799,059	24,799,757	5	9.45 × 10^−5^	0.0199	0.70	0.1293	20.0%
*SLC25A16*	chr10	70,287,181	70,287,493	6	1.07 × 10^−5^	0.0216	−0.45	0.4813	0.0%
*EPHA2*	chr1	16,482,553	16,483,528	8	1.09 × 10^−4^	0.0216	0.47	0.4470	0.0%
*TNFSF13*	chr17	7,460,690	7,462,249	12	1.32 × 10^−4^	0.0223	0.65	0.1888	8.3%
*UBASH3A*	chr21	43,822,540	43,823,863	6	1.69 × 10^−4^	0.0252	−0.64	0.1916	0.0%
*C7orf49*	chr7	134,852,662	134,855,381	9	1.74 × 10^−4^	0.0256	0.59	0.2703	11.1%
*ADAP2*	chr17	29,247,612	29,248,848	9	1.95 × 10^−4^	0.0266	−0.39	0.5675	0.0%
*AMT*	chr3	49,459,855	49,461,563	11	1.97 × 10^−4^	0.0266	0.37	0.5997	0.0%
*C9orf47*	chr9	91,604,473	91,606,140	12	2.64 × 10^−4^	0.0318	0.79	0.0518	18.2%
*CD3D*	chr11	118,213,272	118,214,927	8	2.78 × 10^−4^	0.0318	−0.20	0.8262	0.0%
*HSPB6*	chr19	36,247,867	36,248,907	8	2.82 × 10^−4^	0.0318	0.24	0.7811	0.0%
*STAB1*	chr3	52,528,714	52,529,393	8	3.04 × 10^−4^	0.0329	0.79	0.0534	12.5%
*ACY3*	chr11	67,415,183	67,418,365	8	3.26 × 10^−4^	0.0329	0.72	0.1045	62.5%
*MPG*	chr16	125,896	128,009	11	3.29 × 10^−4^	0.0329	0.82	0.0368	10.0%
*KLRD1*	chr12	10,455,788	10,460,639	8	3.34 × 10^−4^	0.0329	−0.73	0.1030	12.5%
*FES*	chr15	91,427,184	91,428,456	10	3.50 × 10^−4^	0.033	0.39	0.5737	0.0%
*ESM1*	chr5	54,281,198	54,282,459	13	3.97 × 10^−4^	0.036	0.79	0.0526	61.5%
*CTSG*	chr14	25,045,625	25,046,267	6	4.28 × 10^−4^	0.0382	−0.36	0.6151	0.0%
*HMGN2*	chr1	26,797,576	267,987,40	6	4.71 × 10^−4^	0.0403	−0.45	0.4827	0.0%
*LRG1*	chr19	4,540,003	4,540,782	6	4.71 × 10^−4^	0.0403	−0.42	0.5212	0.0%
*LOC100130933*	chr17	73,641,809	73,642,991	10	4.81 × 10^−4^	0.0405	0.44	0.5036	0.0%
*HMGCR*	chr5	74,632,477	74,637,028	5	5.10 × 10^−4^	0.0419	0.71	0.1165	20.0%
*RPSAP52*	chr12	66,220,754	66,221,950	6	5.14 × 10^−4^	0.0419	−0.77	0.0683	0.0%
*MIR145*	chr5	148,808,721	148,810,180	7	5.18 × 10^−4^	0.0419	−0.38	0.5867	0.0%
*PILRA*	chr7	99,970,448	99,971,016	5	5.31 × 10^−4^	0.0419	0.37	0.6031	0.0%
*TMEM219*	chr16	29,972,752	29,974,294	6	5.57 × 10^−4^	0.0431	0.77	0.0667	66.7%
*GIMAP1*	chr7	150,412,503	150,415,143	5	5.86 × 10^−4^	0.0451	0.33	0.6550	0.0%
*CREBBP*	chr16	3,930,112	3,931,489	5	6.16 × 10^−4^	0.0457	0.76	0.0725	80.0%
*MAST2*	chr1	46,268,158	46,269,120	6	6.21 × 10^−4^	0.0457	0.70	0.1248	16.7%
*TAGLN3*	chr3	111,717,534	111,718,245	8	6.29 × 10^−4^	0.0457	0.68	0.1531	12.5%
*S100P*	chr4	6,694,923	6,695,698	9	6.30 × 10^−4^	0.0457	−0.51	0.3861	0.0%
*FAM53C*	chr5	137,672,901	137,675,418	7	6.48 × 10^−4^	0.0458	−0.25	0.7705	0.0%
*GAMT*	chr19	1,401,372	1,402,626	5	6.58 × 10^−4^	0.0459	−0.35	0.6314	0.0%
*H6PD*	chr1	9,293,583	9,303,499	10	6.77 × 10^−4^	0.0462	0.39	0.5714	0.0%
*ITGAE*	chr17	3,704,471	37,058,75	10	6.77 × 10^−4^	0.0462	−0.45	0.4790	0.0%
*CDH9*	chr5	27,036,352	27,040,099	10	6.77 × 10^−4^	0.0462	−0.42	0.5366	0.0%
*ELP2*	chr18	33,709,151	33,709,799	7	6.91 × 10^−4^	0.0462	−0.22	0.8067	0.0%
*LRP11*	chr6	150,185,188	150,186,488	8	7.16 × 10^−4^	0.0462	0.54	0.3423	12.5%
*FIGLA*	chr2	71,017,541	71,018,823	11	7.46 × 10^−4^	0.0473	0.61	0.2324	18.2%
*CAPN13*	chr2	31,020,802	31,031,755	6	8.07 × 10^−4^	0.0496	0.48	0.4419	0.0%

* *BCL2L2-PABPN1,* Maximum Bone Correlation = maximum correlation coefficient representing strength of correlation between blood and bone CpG sites across all sites within the DMR (from Ebrahimi et al.), FDR *p* Value For Maximum Bone Correlation = FDR adjusted *p* value for maximum correlation coefficient across all sites included in the DMR (from Ebrahimi et al.), Percent Positively Strongly Correlated Probes Across DMR = Percentage of probes across the entire region where the correlation coefficient representing strength of correlation between blood and bone CpG sites is greater than 75th percentile among all probes tested in Ebrahimi et al.

**Table A3:** Based on 58 DMRs for Discordant Curve Severity, one Significantly Enriched Ontology was Identified. Analysis of Discordant Curve Severity in Twin Pairs Identified n = 57 Differentially Methylated Regions where Hyper- or Hypomethylation was Consistent Across all Probes in the Region.

Category	Fold Enrichment	Bonferroni Adjusted *p* Value
Gene Ontology Cellular Component		
secretory granule lumen (GO:0034774)	3.8	7.29 × 10^−3^

**Table A4:** Analysis of Curve Severity in Twin Pairs Identified 28 Differentially Methylated Regions where Slope Representing Association between Difference in Methylation and Difference in Curve Severity within each Twin Pair was Consistent Across all Probes in the Region.

Feature/Gen	Chr.	Start Position	End Position	Number of Probes	Nominal *p* Value	FDR *p* Value	Maximum Bone Correlation	FDR *p* Value for Maximum Bone Correlation	Percent Strongly Positively Correlated Probes Across DMR
*NNAT*	chr20	36,148,133	36,149,750	34	1.11 × 10^−5^	0.0237	0.57	0.2898	12.1%
*TMEM232*	chr5	110,021,543	110,062,837	11	5.09 × 10^−5^	0.0237	0.68	0.1441	30.0%
*SLC22A20*	chr11	64,979,837	64,981,596	9	8.43 × 10^−5^	0.0252	0.66	0.1680	33.3%
*PDE12*	chr3	57,541,377	57,543,243	5	9.89 × 10^−5^	0.0252	0.69	0.1347	40.0%
*SUV420H2*	chr19	55,850,082	55,852,507	5	1.98 × 10^−4^	0.0299	0.70	0.1324	40.0%
*CYR61*	chr1	86,045,347	86,046,661	10	2.22 × 10^−4^	0.0299	0.71	0.1148	40.0%
*ZNF440*	chr19	11,924,860	11,925,219	7	2.63 × 10^−4^	0.0333	0.63	0.2110	14.3%
*LOC150622*	chr2	6,072,139	6,072,801	5	3.16 × 10^−4^	0.0348	0.61	0.2383	40.0%
*GANC*	chr15	42,565,522	42,566,390	7	3.38 × 10^−4^	0.0357	0.88	0.0153	28.6%
*TMEM87A*	chr15	42,565,522	42,566,390	7	3.38 × 10^−4^	0.0357	NA	NA	NA
*NME3*	chr16	1,821,559	1,822,346	8	3.59 × 10^−4^	0.0366	0.76	0.0729	37.5%
*SLC6A5*	chr11	20,619,598	20,621,109	8	3.59 × 10^−4^	0.0366	0.80	0.0489	28.6%
*HSPB6*	chr19	36,247,867	36,248,907	8	3.98 × 10^−4^	0.0378	0.24	0.7811	0.0%
*RAB22A*	chr20	56,883,532	56,885,003	8	4.38 × 10^−4^	0.0391	0.78	0.0594	25.0%
*SLC1A1*	chr9	4,489,544	4,490,288	6	4.60 × 10^−4^	0.0394	0.60	0.2551	16.7%
*ACTN4*	chr19	39,137,911	39,138,334	7	4.89 × 10^−4^	0.0407	0.84	0.0298	33.3%
*PRKD1*	chr14	30,396,845	30,397,763	6	4.96 × 10^−4^	0.0407	0.49	0.4133	16.7%
*STL*	chr6	125,284,212	125,284,659	6	4.96 × 10^−4^	0.0407	0.22	0.8030	0.0%
*CPXM1*	chr20	2,781,122	2,782,348	9	5.06 × 10^−4^	0.0411	0.43	0.5202	0.0%
*INSR*	chr19	7,294,087	7,295,192	5	5.27 × 10^−4^	0.0413	0.61	0.2438	20.0%
*NPY*	chr7	24,322,873	24,324,570	8	5.58 × 10^−4^	0.0421	0.84	0.0276	37.5%
*RAB38*	chr11	87,908,558	87,909,729	9	6.33 × 10^−4^	0.045	0.73	0.0995	44.4%
*CNTNAP5*	chr2	124,782,117	124,783,254	9	6.33 × 10^−4^	0.045	0.63	0.2146	11.1%
*AKR7L*	chr1	19,600,471	19,601,069	7	7.52 × 10^−4^	0.0495	0.49	0.4219	0.0%
*COPB1*	chr11	14,521,639	14,522,617	6	7.79 × 10^−4^	0.0495	0.88	0.0149	50.0%
*MEI1*	chr22	42,095,347	42,095,536	5	7.91 × 10^−4^	0.0495	0.65	0.1891	40.0%
*PPP2R1B*	chr11	111,637,044	111,638,422	5	7.91 × 10^−4^	0.0495	0.69	0.1381	40.0%
*KCNB1*	chr20	48,098,642	48,100,238	8	7.97 × 10^−4^	0.0495	0.55	0.3315	12.5%

Maximum Bone Correlation = maximum correlation coefficient representing strength of correlation between blood and bone CpG sites across all sites included in the DMR (from Ebrahimi et al.), FDR *p* Value for Maximum Bone Correlation = FDR adjusted *p* value for maximum correlation coefficient across all sites included in the DMR (from Ebrahimi et al.), Percent Strongly Positively Correlated Probes Across DMR = Percentage of probes across the entire region where the correlation coefficient representing strength of correlation between blood and bone CpG sites is greater than 75th percentile among all probes tested in Ebrahimi et al., NA = probes unavailable.

**Table A5:** Significantly Enriched Ontologies Based on Curve Severity DMR Promoter Analysis.

Category	Fold Enrichment	Bonferroni Adjusted *p* Value
Gene Ontology Molecular Function		
RNA polymerase II cis-regulatory region sequence-specific DNA binding (GO:0000978)	2.4	1.27 × 10^−3^
DNA-binding transcription factor activity, RNA polymerase II-specific (GO:0000981)	2.3	3.65 × 10^−4^
regulatory region nucleic acid binding (GO:0001067)	2.2	1.04 × 10^−3^
transcription regulator activity (GO:0140110)	1.9	2.04 × 10^−2^
Gene Ontology Cellular Component		
chromatin (GO:0000785)	2.3	7.60 × 10^−3^
Gene Ontology Biologic Process		
pituitary gland development (GO:0021983)	12.0	8.09 × 10^−3^
anterior/posterior pattern specification (GO:0009952)	5.2	3.91 × 10^−3^
mesenchyme development (GO:0060485)	4.8	8.84 × 10^−3^
heart morphogenesis (GO:0003007)	4.4	2.44 × 10^−3^
embryonic organ development (GO:0048568)	3.3	4.70 × 10^−3^
negative regulation of cell differentiation (GO:0045596)	3.0	6.46 × 10^−3^
head development (GO:0060322)	2.7	2.37 × 10^−3^
tube development (GO:0035295)	2.7	3.04 × 10^−3^
neuron differentiation (GO:0030182)	2.5	5.09 × 10^−3^
anatomical structure morphogenesis (GO:0009653)	2.0	3.30 × 10^−3^
